# Sucrose- and fat-related metabolic states influence the adaptation of the pulmonary lipid metabolism to hypoxia

**DOI:** 10.1007/s00441-025-03968-0

**Published:** 2025-04-01

**Authors:** Sophia Pankoke, Lea Gerling, Matthias Ochs, Christian Mühlfeld, Julia Schipke

**Affiliations:** 1 Institute of Anatomy, University of Veterinary Medicine, Hannover, Germany; 2 Hannover Medical School, Institute of Functional and Applied Anatomy, Carl-Neuberg-Straße 1, 30625 Hannover, Germany; 3 Institute of Functional Anatomy, Charité – Universitätsmedizin Berlin, Berlin, Germany; 4 German Center for Lung Research (DZL), Berlin, Germany; 5 German Center for Lung Research (DZL), BREATH, Hannover, Germany

**Keywords:** Dietary fat, Dietary carbohydrates, Lipid metabolism, Nutrition, Obesity, Proteomics, Pulmonary surfactant

## Abstract

Pulmonary surfactant is essential for lung function and consists mainly of lipids, almost half of which in adult mammals originate from de novo synthesis in alveolar epithelial type-2 (AE2) cells. Obesogenic nutrition and hypoxia coexist in obese patients with chronic lung diseases. This study tested the hypothesis that diet-induced obesity and chronic hypoxia alter lipid metabolism and thereby deteriorate surfactant homeostasis. Male C57BL/6N mice were fed control diet (4% fat, 6% sucrose; CD), high-sucrose diet (4% fat, 46% sucrose; HSD) or high-fat diet (35% fat, 7% sucrose; HFD). After 27 weeks, half of each diet group was exposed to hypoxia (13% O_2_, Hyp) for 3 weeks. After 30 weeks, lung mechanics were assessed, and the blood, livers, and lungs were analyzed. In CD-fed mice, hypoxia induced lung mechanical changes indicative of reduced elastic recoil properties, as well as smaller lamellar bodies (LBs) and higher composite body volumes, suggesting an increased surfactant precursor formation. HSD and HFD induced lipid accumulation in liver and AE2 cells. In HSD-Hyp and HFD-Hyp, LB volumes per alveolar surface area were elevated, indicating compensatory increases in intracellular surfactant pools which were absent in CD-Hyp. Additionally, hypoxia-related lung mechanics alterations were less pronounced in HSD-Hyp and HFD-Hyp. Lung proteome analysis revealed that only a few lipid metabolism-associated proteins were similarly regulated within diet groups under hypoxia, with the most prominent changes in sucrose-fed hypoxic animals. Thus, individual diet-related metabolic states specifically affect the adaptation of the pulmonary lipid metabolism and intracellular surfactant assembly to chronic hypoxia.

**Supplementary Information:**

The online version contains supplementary material available at 10.1007/s00441-025-03968-0.

## Introduction

Lipids play a crucial role in cell homeostasis as they serve as energy source, constitute structural components of cellular membranes, and are involved in signal transduction. In the lung, lipids have an additional function as the main component of surfactant, which is essential for organ function. Pulmonary surfactant reduces the surface tension at the air–liquid interface within alveoli, preserves pulmonary fluid balance, and acts as an immunological defense system against inhaled pathogens, and is thus essential for lung function (Clements and Avery 1998; Possmayer et al. 2023). Mammalian surfactant consists of ~ 80% phospholipids (primarily phosphatidylcholine, PC), ~ 5 to 10% neutral lipids (mainly cholesterol), and ~ 10% proteins (Possmayer et al. 2023). Alveolar epithelial type-2 (AE2) cells are the sites of synthesis, storage, secretion, and recycling of surfactant (Orgeig et al. 2015). Intracellular surfactant pools are stored in lamellar bodies (LBs). Multivesicular bodies (MVBs, lucent and dark forms) are involved in surfactant uptake from the alveolar air space for recycling or degradation, and in shuttling of surfactant components from cellular synthesis sites to the forming lamellar bodies. Composite bodies (CBs) are MVBs with lamellae that represent an immature form of lamellar bodies (Orgeig et al. 2015; Williams 1987).

In adult mammals, ~ 45% of surfactant PC originates from de novo synthesis within AE2 cells (Batenburg 1992). Only little is known about the provision of synthetic precursors for surfactant lipid synthesis. Biochemically, the fatty acid (FA) moiety of phospholipids may be derived from either circulating free FA or from FAs that are synthesized de novo. It has been shown that de novo FA synthesis or lipogenesis occurs primarily in the liver and adipose tissue (Ameer et al. 2014), but also within the lung (Maniscalco et al. 1989). The interaction between these different sources of pulmonary FAs is incompletely understood, and it may be influenced by the concentrations of accessible substrates, nutritional factors, and distinct physiological states.

In animal models, the composition of surfactant is responsive to circulating lipid concentrations (Fessler and Summer 2016), and in healthy volunteers, a high body mass index is correlated with increased amounts of surfactant lipids (Brandsma et al. 2018). Moreover, the intake of carbohydrates or fat differentially affects surfactant lipid composition and function in mice (Schipke et al. 2021).

Another condition influencing lipid homeostasis is hypoxia. Alveolar hypoxia occurs at high altitudes or due to specific chronic lung diseases. Under hypoxia, cells switch from an oxidative to an anaerobic glycolytic metabolism for energy production. Thus, the conversion of the glycolytic end product pyruvate to Acetyl-CoA, which normally feeds the citric acid cycle and is transformed to citrate, is blocked. As a consequence, Acetyl-CoA as a precursor for de novo FA synthesis is missing. Moreover, FA catabolism by aerobic β-oxidation is impaired, and many cells store the surplus of FAs in the form of lipid droplets (LDs) to avoid lipotoxicity (Mylonis et al. 2012). In cultivated murine AE2 cells, hypoxia results in a shift to a glycolytic phenotype, which is accompanied by an altered expression of enzymes implicated in lipid synthesis and trafficking (Lottes et al. 2014). Furthermore, chronic hypoxia in rats leads to an increase in inflammation-related proteins and hemoglobin in the lungs, as well as to a slightly increased surface activity of the isolated surfactant with a constant expression of surfactant proteins (Olmeda et al. 2014).

Given the increasing prevalence of obesity and chronic lung diseases, the simultaneous occurrence of dyslipidemia and alveolar hypoxia is a common scenario in patients. However, little is known about how the combination of an obesogenic diet and chronic hypoxia affects pulmonary lipid homeostasis and surfactant production in AE2 cells. A previous study showed that hypoxia-induced changes of the lung proteome and pulmonary ultrastructure differed significantly between control diet- and high-fat diet-fed mice (Pankoke et al. 2023), indicating a nutritional effect on the adaptation of the lung to hypoxia.

This study tested the hypothesis that differences in metabolic states induced by diet (high-sucrose intake or high-fat intake) and chronic hypoxia affect the lipid metabolism of the lung and, as a consequence, the surfactant synthesis in AE2 cells.

## Material and methods

### Animal experiments

Animal experiments were approved by the Lower Saxony State Office for Consumer Protection (LAVES, file number 18/2841) and conducted in accordance with the European Directive 2010/63/EU. Male C57BL/6N mice were purchased from Charles River (Sulzfeld, Germany) at an age of 5 weeks. After one week of acclimatization, mice were randomly allocated to one diet group, either a purified control diet (CD; 4% fat, 6% sucrose; S3542-E040, ssniff Spezialdiäten, Soest, Germany), purified high-sucrose diet (HSD; 4% fat, 46% sucrose; S3542-E042, ssniff) or purified high-fat diet (HFD; 35% fat, 7% sucrose; S3542-E044, ssniff). For the detailed composition of the diets, please refer to Pankoke et al. (2022), Table 1 (Pankoke et al. 2022). Mice had ad libitum access to food and water, and were housed individually in cages equipped with shelters and nesting material under temperature-controlled conditions (21 ± 2 °C). After 27 weeks, half of each diet group was subjected to normobaric hypoxia (13% O_2_, Hyp) in a Plexiglas chamber (BioSpherix, Ltd., Parish, NY, USA) for 3 weeks, resulting in the following groups: CD, CD-Hyp, HSD, HSD-Hyp, HFD, and HFD-Hyp (*n* = 6). This group size was based on an *F*-test, one-way ANOVA, a priori power analysis with a critical significance level of 0.05, a power of 0.80, and an effect size of 0.7. In week 29, mice were fasted for 6 h, and retroorbital blood was collected for plasma analyses.

**Table 1:** Systemic effects of diet and hypoxia

Diet	Control diet (CD)		High-sucrose diet (HSD)		High-fat diet (HFD)	
**Exposure**	**Normoxia**	**Hypoxia**	**Normoxia**	**Hypoxia**	**Normoxia**	**Hypoxia**
**Body weight** (g)	37.4 ± 3.4	36.1 ± 3.7	34.6 ± 2.9	33.3 ± 3.5	49.2 ± 4.1 diet: *p* < 0.001	41.6 ± 3.4 diet: *p* = 0.028 hyp: *p* = 0.001
**Arterial hematocrit** (%PCV)	36.2 ± 1.8	43.2 ± 2.1 hyp: *p* < 0.001	37.8 ± 2.0	43.5 ± 1.9 hyp: *p* = 0.003	36.3 ± 2.8	44.0 ± 5.5 hyp: *p* < 0.001
**Arterial hemoglobin** (g/dL)	12.3 ± 0.6	14.7 ± 0.7 hyp: *p* < 0.001	12.9 ± 0.7	14.8 ± 0.6 hyp: *p* = 0.003	12.4 ± 1.0	15.0 ± 1.9 hyp: *p* < 0.001
**Tunica media thickness of peripheral pulmonary arteries** (µm)	5.4 ± 0.8	9.2 ± 0.6 hyp: *p* < 0.001	6.1 ± 1.2	8.4 ± 1.6 hyp: *p* = 0.017	5.9 ± 1.2	10.0 ± 2.4 hyp: *p* < 0.001
**Retroperitoneal WAT** (g)	0.63 ± 0.25	0.51 ± 0.17	0.49 ± 0.10	0.44 ± 0.16	1.19 ± 0.39 diet: *p* = 0.003	0.75 ± 0.19 hyp: *p* = 0.02
**Interscapular WAT/BAT** (g)	0.36 ± 0.09	0.28 ± 0.08	0.35 ± 0.08	0.28 ± 0.05	0.82 ± 0.29 diet: *p* < 0.001	0.42 ± 0.09 diet: *p* = 0.019 hyp: *p* < 0.001
**Total cholesterol** (mg/dL)	93.0 ± 13.4	78.7 ± 17.9	119.7 ± 20.2	97.0 ± 20.3	153.7 ± 30.8 diet: *p* < 0.001	150.7 ± 28.1 diet: *p* < 0.001
**HDL-cholesterol** (mg/dL)	78.0 ± 10.2	65.0 ± 17.4	98.3 ± 15.8	75.0 ± 11.9 hyp: *p* = 0.035	122.0 ± 25.3 diet: *p* < 0.001	114.3 ± 23.8 diet: *p* < 0.001
**LDL-cholesterol** (mg/dL)	10.7 ± 5.5	7.5 ± 2.7	20.0 ± 7.7 diet: *p* = 0.024	12.7 ± 3.5 hyp: *p* = 0.037	28.7 ± 5.9 diet: *p* < 0.001	25.0 ± 7.7 diet: *p* < 0.001
**Triglycerides** (mg/dL)	57.7 ± 6.0	73.2 ± 18,5 hyp: *p* = 0.031	59.0 ± 2.1	67.7 ± 12.1	72.3 ± 13.2	75.3 ± 11.6
**Glucose** (mmol/L)	9.6 ± 1.1	8.6 ± 2.6	10.2 ± 0.9	8.7 ± 2.0	10.0 ± 1.6	9.6 ± 0.9
**Insulin** (ng/mL)	0.86 ± 0.35	0.88 ± 0.46	0.72 ± 0.37	0.96 ± 0.47	3.42 ± 1.50 Diet: *p* = 0.002	2.67 ± 1.61 Diet: *p* = 0.027

Values are presented as mean ± SD. Statistics: two-way ANOVA (factors diet and O_2_) and pairwise comparisons by post-hoc Tukey’s test; significant diet effects = *p* < 0.05 vs. CD at the same O_2_ is indicated by “diet: *p*-value”; significant hypoxia effects = *p* < 0.05 vs. normoxia in the same diet group is indicated by “hyp: *p*-value”

*WAT* white adipose tissue, *BAT* brown adipose tissue, *HDL* high-density lipoprotein, *LDL* low-density lipoprotein

After 30 weeks, mice were anesthetized (100 mg/kg body weight ketamine, 5 mg/kg body weight xylazine, i.p.), and lung mechanics were assessed under closed chest conditions. After control of adequate analgesia and muscle relaxation, measurements were performed at a positive end-expiratory pressure of 3 cmH_2_O using a FlexiVent small animal respirator (SQIREQ, Montreal, QC, Canada). Arterial blood from the tail artery was used for the analysis of hemoglobin and hematocrit with the iSTAT Alinity device (CG8 + cartridges; Abbott, Wiesbaden, Germany). For harvesting of the lung, the thoracic cage was opened, and the right lung lobes were ligated and snap-frozen in liquid nitrogen for proteome analysis, whereas the left lungs were instillation-fixed with 1.5% paraformaldehyde/1.5% glutaraldehyde/0.15 M HEPES buffer at a hydrostatic pressure of 20–25 cmH_2_O for structural analyses. Livers were isolated and immersion-fixed in 4% paraformaldehyde/0.1 M phosphate buffer and post-fixed in 1.5% paraformaldehyde/1.5% glutaraldehyde/0.15 M HEPES. The animals presented in this study are part of a larger animal cohort. Of some animals, body weights, fat depot weights, plasma values, arterial hemoglobin and hematocrit values, tunica media thickness of peripheral pulmonary arteries, hepatic lipid accumulation, proteome data, and lung mechanical parameters were shown previously but either not in the context of hypoxia (Pankoke et al. 2022) or not in comparison to sucrose-fed mice (Pankoke et al. 2023).

### Plasma analysis

Mice were food-deprived for 6 h, retro-orbital blood was collected, and blood plasma was isolated by centrifugation. Plasma concentrations of lipids and glucose were analyzed in the Institute for Clinical Chemistry, Hannover Medical School, using Cholesterol Gen.2, HDL Cholesterol plus third-generation, LDL Cholesterol Gen.3, and TRIGL kits according to the manufacturer’s instructions (Roche Diagnostics, Mannheim, Germany) together with a Cobas c automated analyzer system (Roche/Hitachi). Insulin levels were assessed in duplicates using an ultrasensitive mouse insulin ELISA (#90080, Chrystal Chem, Elk Grove Village, IL, USA).

### Proteome analysis

#### Protein isolation and digestion

Snap-frozen lung tissue was homogenized in RIPA buffer containing protease inhibitors (Merck, Darmstadt, Germany; Roche Diagnostics, Mannheim, Germany). After sonication on ice, lysates were centrifuged (10 min, 4 °C, 25,000 × *g*) and protein contents of supernatants were determined by the PierceTM BCA Kit (Thermo Fisher Scientific, Waltham, MA, USA). 50 µg protein in 4 × Laemmli (Bio-Rad, Hercules, CA, USA) was added with mercaptoethanol (Sigma-Aldrich; St. Louis, MO, USA), incubated for 10 min at 70 °C, and alkylated with 1 µl of acrylamide (40%) at room temperature for 30 min. Proteins were separated on gradient gels (anykD gel; Bio-Rad) by SDS-PAGE (Sodium Dodecyl Sulfate–Polyacrylamide Gel Electrophoresis), gels were stained with Page Blue (Thermo Fisher Scientific), and gel lanes were cut into 1 × 1 × 1 mm cubes. Cubes were destained with 50% acetonitrile (ACN) and 20 mM ammonium bicarbonate (ABC) for in-gel digestion. This was followed by dehydration with 100% ACN and vacuum centrifugation. The dried cubes were rehydrated for 60 min on ice using 10% ACN/20 mM ABC containing 10 ng/µL trypsin (Promega, Madison, WI, USA), and were subsequently incubated overnight at 350 rpm and 37 °C. By adding 100 µl 50% ACN/5% trifluoroacetic acid (TFA) for 30 min, the protein digestion was stopped, and the peptides were extracted with 100 µl 50% ACN/0.5% TFA for 30 min and 100 µl 100% ACN for 20 min at 450 rpm. After the pooling of the supernatants, the extracted peptides were dried by vacuum centrifugation and stored at −20 °C.

#### *Peptide analysis by LC*–*MS*

For the analysis, an LTQ Orbitrap Velos mass spectrometer (Thermo Fisher Scientific) was equipped with a nano-electrospray source and connected to an Ultimate 3000 RSLC nanoflow system (Thermo Fisher Scientific). The dried peptides were dissolved in 0.1% TFA/2% CAN and loaded onto a C18 PepMap100 (5 µm, 100 Å; Thermo Fisher Scientific) µ-precolumn before separation in a 50 cm µPACTM analytical column (Thermo Fisher Scientific) at 35 °C. A binary gradient was used to separate the peptides, consisting of solvent A (0.1% formic acid; Merck) and solvent B (80% ACN/0.1% formic acid; Merck). The column was prepared with 4% of solvent B for 15 min, followed by an increase of solvent B up to 25% within the next 115 min for equilibration. Within the following 25 min, solvent B was increased up to 50% for further separation before being increased to 90% within 15 min and kept at this level for a further 5 min to remove the highly hydrophobic molecules from the column. Within the next 10 min, solvent B was then decreased to 4% and kept at this percentage for another 15 min for re-equilibration of the column. A spray voltage of 1.6 kV and a data-dependent acquisition approach were used for further measurements. To obtain the final mass spectrometric (MS)2 spectra, the top ten peaks from MS1 measurements performed in the Orbitrap with a resolution of 60,000 (threshold value of 2000 counts) were further fragmented with collision-induced dissociation at a normalized collision energy of 38%. The ion trap for the MS2 measurements was set to an isolation width of 2 m/z and an activation time of 10 ms. On peptides isolated for MS2 measurements, a dynamic exclusion of 70 s was applied.

#### Data processing

For the initial raw data processing MaxQuant software version 1.6.17.0 with the Andromeda search engine was used (Cox and Mann 2008; Cox et al. 2011). The MS spectra were searched against the mouse entries of the Swiss-Prot reviewed UniProtKB database (UP000000589, 55,311 entries) to identify the peptides (Bateman et al. 2021). A maximum of two missed cleavages and a false discovery rate of 0.01 on protein and peptide levels were accepted. Additionally, accepted variable modifications were the oxidation of methionine, N-terminal acetylation, and deamidation of glutamine and asparagine, whereas the propionamidation of cysteine was set as a fixed modification. A minimum ratio count of one razor peptide was required for quantification.

Further protein examination was performed using the Perseus software (version 1.6.14; (Tyanova et al. 2016)). Proteins had to be present in 80% of the animals in at least one experimental group to be included for further analysis. The included data were then log_2_-transformed and normalized by median, and missing values were imputed from the normal distribution.

### Structural analysis

Further left lung processing and structural analyses were performed with design-based stereological methods (Ochs and Schipke 2021) in accordance with the recommendations of the American Thoracic Society and European Respiratory Society (Hsia et al. 2010). The analyst was blinded for experimental groups. The water displacement method (Archimedes Principle) was used for the determination of the left lung volume (Scherle 1970). Subsequently, the lung tissue was sampled with systematic uniform random sampling (SURS; (Ochs and Schipke 2021)). For light microscopy (LM) analysis, lung tissue was embedded in glycol methacrylate (Technovit 7100; Heraeus Kulzer, Wehrheim, Germany) as described previously (Hollenbach et al. 2019). 1.5-µm thick sections were cut, and the first and the third sections were mounted on glass slides resulting in a physical LM disector with a height of 3 µm. Sections were stained with toluidine blue, Sudan Black B and hematoxylin, or orcein and eosin. Sections were digitized using a slide scanner (Axio Scan.Z1, ZEISS, Oberkochen, Germany) at a primary magnification of × 20. For further analysis, the newCast software (Visiopharm, Hørsholm, Denmark) was used.

For electron microscopy (EM) analysis, lung tissue was postfixed in 1% osmium tetroxide, en bloc stained with uranyl acetate and embedded in epoxy resin (Epon; Serva, Heidelberg, Germany) as described before (Hollenbach et al. 2019). Two subsequent 100 nm epoxy resin sections were mounted on copper grids resulting in a physical EM disector with a height of 100 nm and were stained with lead citrate/uranyl acetate. Images were obtained with a transmission electron microscope (Morgagni 268, FEI, Eindhoven, Netherlands). (i) Analysis of AE2 cell organelles: Fields of view were obtained at a primary magnification of × 7100. In the first step, random (according to SURS) AE2 cells were imaged in one section. In the second step, the corresponding AE2 cells were identified and imaged in the other section. (ii) Analysis of septal parameters: Random (according to SURS) images of septa were obtained at a primary magnification of × 14,000 on one section. Three randomly chosen epoxy resin blocks per animal were analyzed using the STEPanizer stereology tool (Tschanz et al. 2011).

For all stereological analyses, SURS methods were used to generate fields of view, which were superimposed with adequate test systems (Ochs and Schipke 2021). The microscopic magnifications were chosen to be high enough for unambiguous identification of structures of interest, but low enough to guarantee efficient analysis. Total volumes were assessed by the point counting method and surface areas were determined by intersection counting as described before (Hollenbach et al. 2019; Ochs and Schipke 2021; Pankoke et al. 2023). The mean volume of AE2 cells was determined by the rotator method, the number of AE2 cells was estimated using the LM disector and counting of nuclei present in the reference section but not in the look-up section. The number of lamellar bodies in AE2 cells was estimated using the EM disector and counting of lamellar body profiles present in the reference section but not in the look-up section (Ochs and Schipke 2021).

Tunica media thickness of pulmonary arteries was measured as the thickness of the region between lamina elastica interna and lamina elastica externa at the largest transverse arterial diameter utilizing the VIS measurement tool (Visiopharm, Hørsholm, Denmark) using orcein-eosin stained sections as described before (Pankoke et al. 2023).

### Hepatic lipid analysis

Liver tissue was postfixed in 1% osmium tetroxide, en bloc stained with uranyl acetate, and embedded in epoxy resin (Epon; Serva, Heidelberg, Germany) as described before (Hollenbach et al. 2019). 1 µm thick sections from three randomly chosen tissue blocks per animal were cut, mounted on glass slides, stained with toluidine blue, and digitized using a slide scanner (Axio Scan.Z1, ZEISS, Oberkochen, Germany) at a primary magnification of × 40. Subsequently, automated image analysis was performed using the Tissuemorph DP software from Visiopharm (version 5.3.1.1723, Hoersholm, Denmark) as described before (Schipke et al. 2022).

### Statistics

Group size calculation was performed with G*Power software (version 3.1.9). For proteome data analysis, an ANOVA was performed using the Perseus software (version 1.6.14). ANOVA-significant proteins were scanned for lipid-metabolism-related proteins according to the Reactome database (https://reactome.org). For the visualization by heat map, an additional *z*-scoring and hierarchical clustering (euclidean distance, 300 clusters, maximum of 10 iterations) was performed. For comparing CD with CD-Hyp, HSD with HSD-Hyp, and HFD with HFD-Hyp, a two-sided two-sample student’s *t*-test was applied. A *p*-value of < 0.05 was considered significantly regulated and the following pathway analysis of group-specific regulated lipid-metabolism-related proteins was performed using the STRING database (version 11.5, https://string-db.org). For data visualization version 9 of GraphPad Prism was used. For all other data, Sigma Plot version 13.0 (Systat Software Inc.) was used to perform two-way ANOVA (tested factors: diet and normoxia/hypoxia) followed by a pairwise comparison by post hoc Tukey test. *p* < 0.05 values were considered statistically significant, and *p* values between 0.05 and 0.1 (0.05 < *p* < 0.1) were considered to show a tendency toward significance (Curran-Everett and Benos 2004). Data are presented as individual values or as mean ± SD. Significant results and tendencies are indicated within figures as stated in figure legends.

## Results

Chronic hypoxia induced increases in arterial hematocrit, arterial hemoglobin, as well as in tunica media thicknesses of peripheral pulmonary arteries irrespective of the diet (Table 1). In HSD-fed normoxic mice, circulating LDL-cholesterol concentrations were increased compared to CD, and HDL- and LDL-cholesterol were significantly decreased under hypoxic conditions in HSD-Hyp compared to HSD. HFD feeding resulted in higher body and fat depot weights under normoxia, which were significantly lowered under hypoxia in HFD-Hyp compared to HFD. Circulating total, HDL- and LDL-cholesterol concentrations were elevated in HFD-fed mice independent of the oxygen supply, and circulating triglyceride concentrations were elevated only in hypoxic, CD-fed mice compared to normoxic controls.

**Fig. 1:**
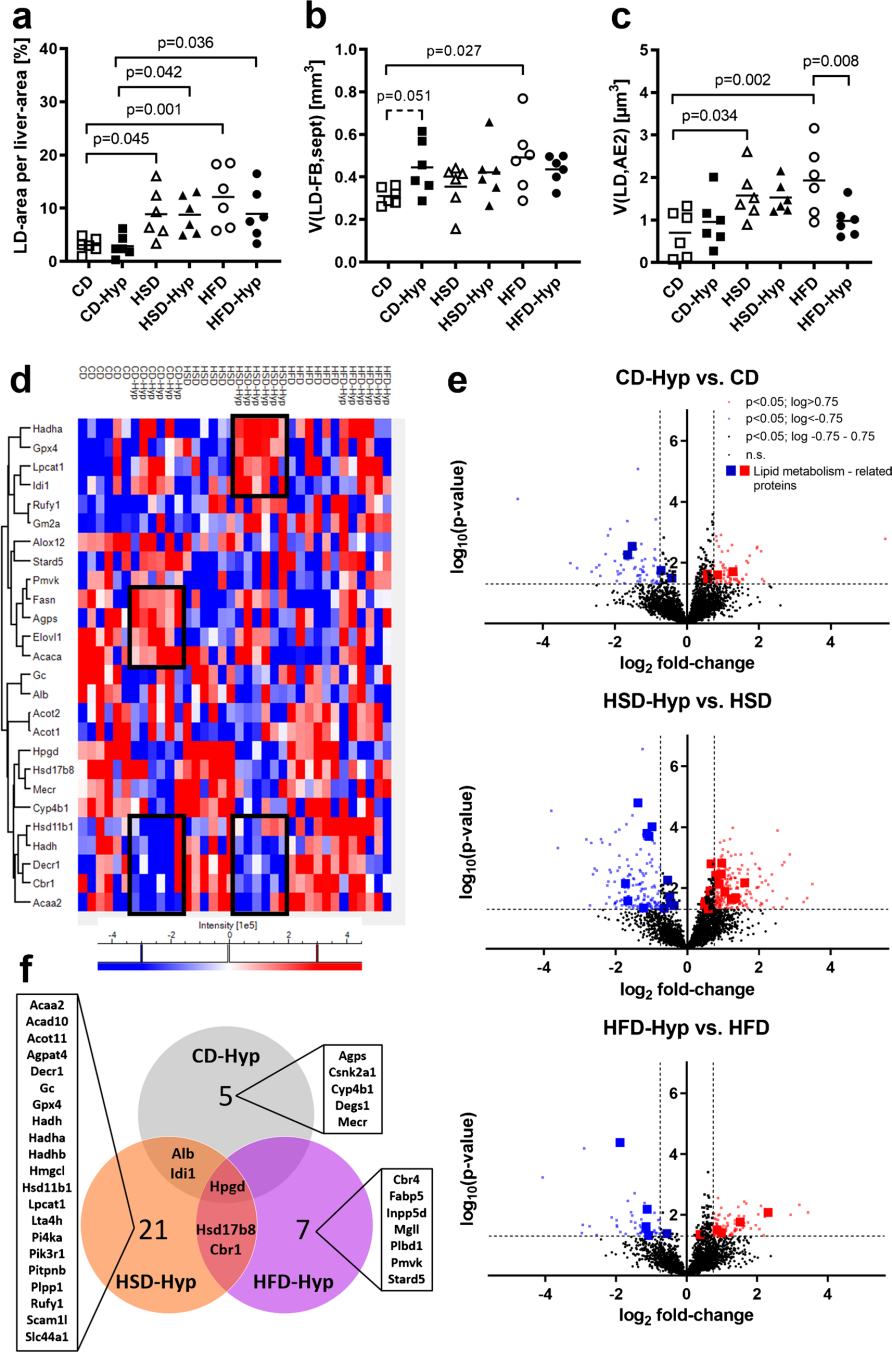
Lipid homeostasis. **a** Volume density of hepatic lipid droplets. **b** Lipid droplet volumes in septal fibroblasts. **c** Lipid droplet volumes in AE2 cells. **d** Heat map of significantly regulated lipid metabolism-associated proteins after multi-sample test ANOVA, hierarchical clustering and *z*-scoring. **e** Volcano plots of significantly regulated proteins according to Student’s t-test. **f** Venn diagram of significantly regulated lipid-metabolism-associated proteins under hypoxia referring to the respective normoxic diet group. Symbols represent individual mice. **a**–**d** Significances and tendencies according to two-way ANOVA and post hoc Tukey test are indicated by *p*-values; **d**–**f** group sizes, *n* = 6

Both, the HSD and the HFD induced hepatic lipid accumulation irrespective of the oxygen supply (Fig. 1a). Within the lung, HFD-feeding was associated with lipid accumulation in both alveolar septal fibroblasts and AE2 cells compared to CD-fed animals, whereby the AE2 cell LD volume was reduced under hypoxic conditions (Fig. 1b and c). In contrast, the HSD was associated with higher LD volumes only in AE2 cells without obvious hypoxia-related effects. In CD-fed animals, the LD volume in fibroblasts showed a trend to increase upon hypoxia, although this did not reach statistical significance. LD volumes in AE2 cells were unaltered in CD-Hyp compared to CD. AE2 cells also contained LDs with lamellae, which were previously reported to accumulate in response to cellular stress, e.g., ischemia (Ochs et al. 2004). These were quantified separately and did not show significant changes in response to diets or hypoxia (Fig. S1).

Lung proteome analysis revealed 387 proteins which showed a significantly altered abundance due to the different diets or hypoxia. A corresponding pathway analysis with STRING-db (https://string-db.org) revealed 125 regulated pathways, several of which were metabolism-related (Table S1). According to the Reactome database (https://reactome.org), 26 of the 387 significantly altered pulmonary proteins were linked to lipid metabolism, with certain protein clusters particularly affected by the specific diets and/or hypoxia (Fig. 1d, rectangles). This was further evaluated by pairwise *t*-tests showing diet-specific differences in hypoxia-related adaptations of the pulmonary lipid metabolism (Fig. 1e). Of the lipid-metabolism-associated proteins (Fig. 1f; Table S2), only one was downregulated in all diet groups due to hypoxia, i.e., 15-hydroxyprostaglandin dehydrogenase (*Hpgd*). Two proteins were altered in both CD-Hyp and HSD-Hyp (serum albumin, *Alb*, and isopentenyl diphosphate delta-isomerase 1, *Idi1*), and two other proteins were altered in both HSD-Hyp and HFD-Hyp (estradiol-17-beta-dehydrogenase 8, *Hsd17b8*, and carbonyl reductase 1, *Cbr1*) compared to the respective normoxic diet groups. Besides this, five proteins were specifically altered in CD-Hyp compared to CD, 21 proteins in HSD-Hyp compared to HSD, and seven proteins in HFD-Hyp compared to HFD. Detailed protein information is given in Supplemental Table S2.

**Fig. 2:**
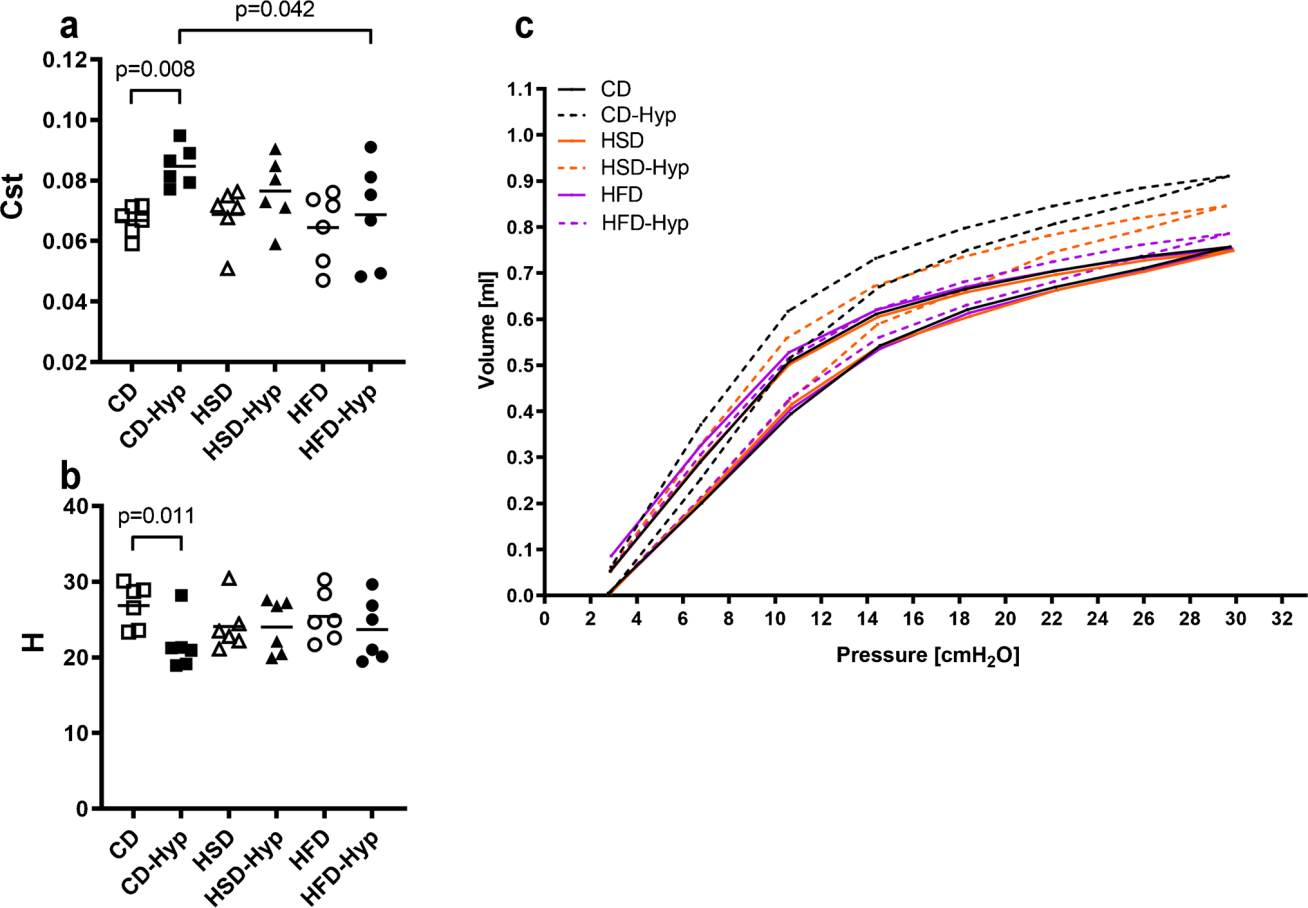
Lung mechanics. **a** Quasi-static lung compliance. **b** Elastance H. **c** Pressure volume curves. **a**–**b** Symbols represent individual mice; **c** group means are shown, group size *n* = 6. **a**–**b** Significances according to two-way ANOVA and post hoc Tukey’s test are indicated by *p*-values

In CD-fed mice, hypoxia induced a significant increase in the quasi-static lung compliance Cst and a decrease in the elasticity H (Fig. 2a and b). Moreover, inspiration limbs of pressure–volume (PV) loops showed a major shift in CD-Hyp (Fig. 2c). These hypoxia-associated lung mechanical alterations were less pronounced or even absent in HSD-Hyp and HFD-Hyp.

**Fig. 3:**
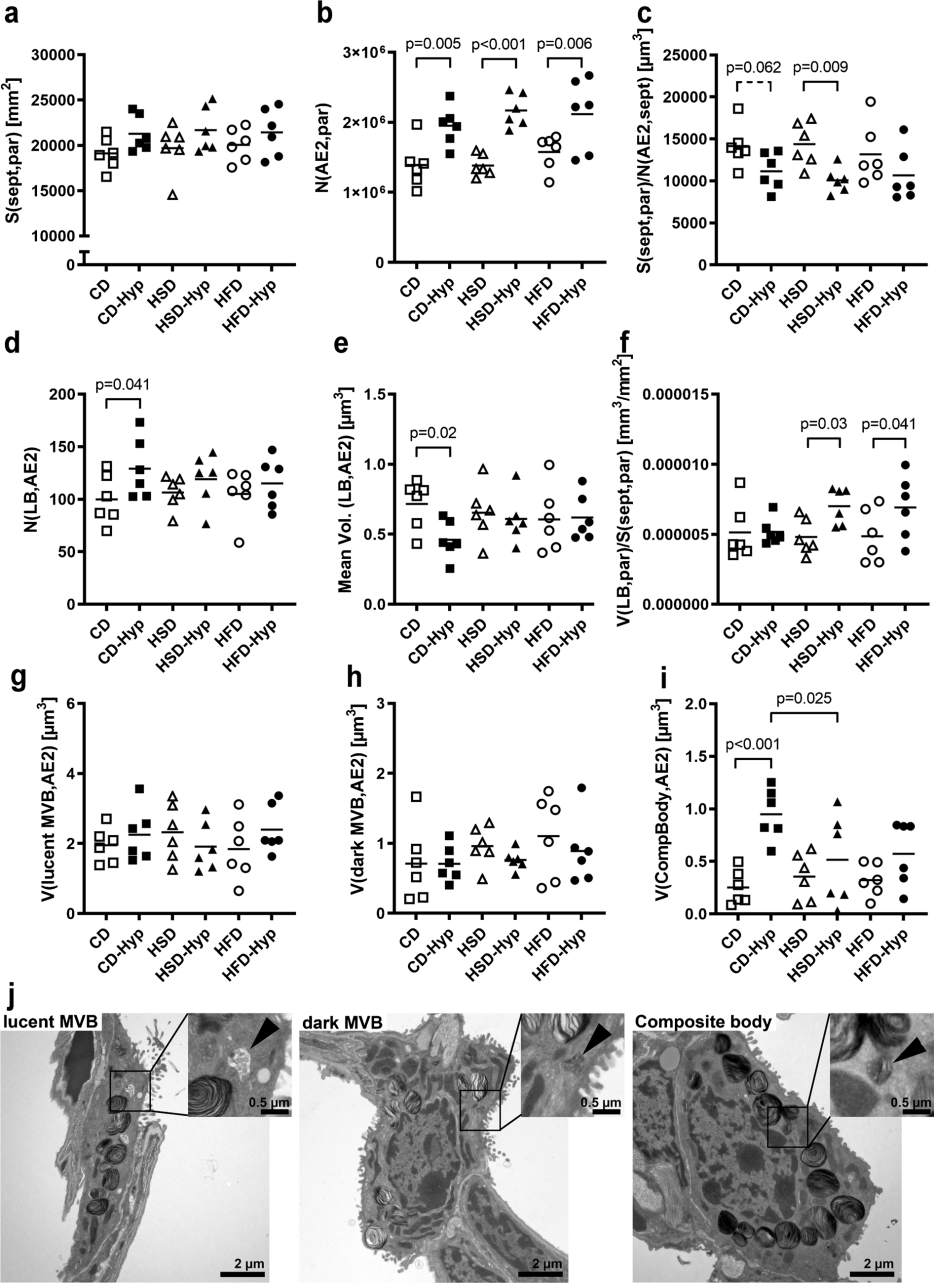
AE2 cells and intracellular surfactant assembly. **a** Alveolar surface area. **b** Number of AE2 cells. **c** Alveolar surface area supplied by one AE2 cell. **d** Number of lamellar bodies per AE2 cell. **e** Mean volume of one lamellar body. **f** LB volume relative to the alveolar surface area. **g** Volume of lucent multivesicular bodies. **h** Volume of dark multivesicular bodies. **i** Volume of composite bodies. **j** Representative electron microscopic images illustrating organelles implicated in surfactant assembly. **a**–**i** Symbols represent individual mice; significances and tendencies according to two-way ANOVA and post hoc Tukey’s test are indicated by *p*-values

Chronic hypoxia-induced an increase in the number of AE2 cells irrespective of the diet (Fig. 3b), although the alveolar surface area remained constant between the experimental conditions (Fig. 3a). As a consequence, the alveolar surface area supplied by one AE2 cell was reduced in HSD-Hyp and, as a tendency, in CD-Hyp groups (Fig. 3c).

In CD-fed mice, chronic hypoxia was associated with a higher number of LBs per AE2 cell and a reduced LB mean volume (Fig. 3d and e). However, LB volumes relative to alveolar surface areas in CD-Hyp were similar to normoxic controls (Fig. 3f). In contrast, in HSD- and HFD-fed mice hypoxia had no effect on the number or mean volume of LBs, which was associated with higher LB volumes per alveolar surface area.

A smaller size of LBs might point to immature lamellar bodies, suggesting an elevated consumption or an impaired synthesis of surfactant. In the CD-Hyp group, the volume of CBs was significantly increased compared to normoxic controls (Fig. 3i). Again, these changes were not observed in hypoxic sucrose- or fat-fed mice. Volumes of electron-lucent and electron-dense MVBs were similar in all groups (Fig. 3g and h).

## Discussion

This study shows that individual diet-related metabolic states influence hypoxia-induced alterations in lipid metabolism and intracellular surfactant assembly. In lean CD-fed mice, hypoxia resulted in elevated circulating triglyceride concentrations, lung mechanical changes indicative of reduced elastic recoil properties, and ultrastructural evidence of increased surfactant precursor formation in AE2 cells. High sucrose or fat intake induced lipid accumulation in AE2 cells, and alleviated hypoxia-related alterations of lung mechanics. Moreover, in HSD-Hyp and HFD-Hyp groups, the volume of LB per septal surface area was significantly higher compared to normoxic controls, indicating a compensatory increase in intracellular surfactant pools that were absent in CD-Hyp animals.

Nutrition and hypoxia are both factors that influence cellular lipid metabolism in different ways. The diet composition determines the type and quantity of lipids that are supplied to the cells via the blood, and a sufficient oxygen supply is necessary for the oxidative breakdown of lipids for energy production and the provision of precursors for de novo lipogenesis. While cells generally use lipids to store energy and maintain cellular membranes, pulmonary AE2 cells additionally need lipids for surfactant synthesis. The exact relative contribution of lipid species provided by the circulation, those supplied by recycling of alveolar phospholipids, and those synthesized de novo to the formation of surfactant lipids are still largely unknown.

The elevated plasma triglyceride levels of the CD-Hyp group are in accordance with previously described disturbing effects of hypoxia on the lipid metabolism, characterized by an overproduction and an impaired clearance of triglyceride-rich lipoproteins resulting in increased plasma triglyceride levels (Morin et al. 2021). This was not the case in HSD- or HFD-fed animals, in accordance with a rodent study showing that intermittent hypoxia does not influence blood lipids in hyperphagic obese animals (Li et al. 2005). The high intake of lard with the HFD led to higher plasma cholesterol levels in the HFD and HFD-Hyp groups.

This differential supply of lipids via the circulation was accompanied by distinct cellular lipid accumulation within the lung. Both, the HSD and the HFD resulted in elevated LD volumes in AE2 cells. This was similar to the lipid accumulation in hepatocytes, which was also observed under both the HSD- and HFD-feeding regimen, possibly pointing to a similar regulation of lipid uptake and storage. In contrast, LD volumes in septal fibroblasts were elevated only in the HFD-group, but not in the HSD-group. The aforementioned increase in circulating triglycerides in CD-Hyp was accompanied by a strong tendency to increased LD volumes in septal fibroblasts. This indicates that not only the supply of circulating lipids but also the cellular lipid metabolism determines lipid accumulation under the analyzed experimental conditions.

The hypoxia-induced adaptation of the pulmonary lipid metabolism was rather different depending on the dietary basis. *Hpgd* was the only protein that was downregulated in response to hypoxia in all diet groups, as reported previously for hypoxic pulmonary hypertension (He et al. 2023) and intermitted hypoxia (Reinke et al. 2011) in mice. *Hpgd* is implicated in prostaglandin catabolism, thereby functioning in a variety of physiological processes such as inflammation and blood pressure homeostasis (Stelling et al. 2020). The latter might be due to a direct effect since transfected *Hpgd* decreases the proliferation, reduces the angiogenesis capacity, and induces apoptosis in isolated lung endothelial cells (He et al. 2023).

In CD-Hyp and HSD-Hyp, i.e., groups without extra fat intake via the diet, *Idi1* was upregulated, and *Alb* was downregulated in the lung. *Idi1* catalyzes an important step during cholesterol synthesis (Chen et al. 2023), and *Alb* functions as an FA transporter in blood and extracellular fluids facilitating the uptake of fatty acids by organs in need of these substrates (Vusse 2009). In HSD-Hyp und HFD-Hyp, i.e., groups with diet-related lipid accumulation in AE2 cells, *Hsd17b8* und *Cbr1* amounts were reduced in the lung. *Hsd17b8* is essential for mitochondrial FA synthesis (Venkatesan et al. 2014), which was shown previously to be altered in obese mice resulting in the accumulation of triglycerides (Zeng et al. 2021). *Cbr1* is an NADPH-dependent reductase with a broad substrate specificity, and knockdown of *Cbr1* increases expression of lipogenic enzymes and intracellular lipid accumulation in pancreatic β-cells (Rashid et al. 2010).

In CD- and HFD-fed mice, hypoxia resulted in expression changes of only a few lipid metabolism-associated proteins in the lung, whereas high sucrose intake combined with hypoxia led to an altered abundance of 26 proteins, indicating a reprogramming of the pulmonary lipid metabolism especially in sucrose-fed mice. The CD diet regimen under hypoxia induced changes in enzymes involved in various aspects of the lipid metabolism. On the one hand, the expression of *Agps* and *Degs1*, which catalyze the synthesis of ether-lipids such as phospholipids and plasmalogens (*Agps*) or ceramide (*Degs1*), a sphingolipid-precursor, was found to be increased. Both plasmalogens and sphingolipids are components of lung surfactants (Kleuser et al. 2023; Zhuo et al. 2021). Consequently, the upregulation of *Agps* and *Degs1* may indicate a modification or increased synthesis of pulmonary surfactant. On the other hand, *Cyp4b1* and *Mecr*, a monooxygenase and a mitochondrial oxidoreductase implicated in fatty acid synthesis, exhibited a decrease in expression in CD-Hyp, which was likely attributable to oxygen deprivation.

Many proteins solely upregulated in HSD-Hyp were implicated in β-oxidation and fatty acid degradation (*Acaa2*, *Acad10*, *Acot11*, *Decr1*) presumably providing energy for hypoxia adaptation. Furthermore, *Lpcat* which catalyzes the synthesis of phosphatidylcholine species—the main surfactant phosphoproteins—was upregulated, indicating an increased production of surfactant precursors (Bridges et al. 2010; Li et al. 2024). HFD-Hyp treatment resulted in altered abundances of proteins involved in lipid degradation (*Mgll*, *Plbd1*) and transport (*Fabp5*, *Stard5*). *Plbd*1 is a phospholipase that degrades various phospholipids, including phosphatidylcholine species. *Fabp5*, an epidermal fatty acid binding protein, is discussed to be implicated in dipalmitoyl–phosphatidylcholine (PC16:0/16:0) synthesis in AE2 cells (Guthmann et al. 2004). Thus, there was an indication of increased energy demand and/or surfactant metabolism under the HFD-Hyp regimen.

As a limitation of the study, the proteome analysis was carried out with bulk lung tissue, thus differences in protein abundances cannot be traced back to individual cell populations.

AE2 cell numbers were increased in response to hypoxia irrespective of the diet pointing to AE2 cell proliferation as an adaptation to low oxygen supply. Since the alveolar surface area was not altered by the experimental conditions, a larger proportion of AE2 cells appears to be required to adequately supply a similar surface area, suggesting abnormalities in surfactant quantity and/or quality. An increase in AE2 cell volumes was also observed in rats exposed to chronic hypoxia, albeit, in these animals, the alveolar surface area increased (Yilmaz et al. 2015). This was attenuated in hyperphagic obese animals, contrary to our data. This discrepancy might be due to the evaluation of total volumes and not cell numbers by Yilmaz et al. (Yilmaz et al. 2015); thus, the observed changes might reflect differences in cell sizes rather than cell numbers.

LBs grow in size along the secretory pathway (Vanhecke et al. 2025). In lean hypoxic mice, more LBs were present which were smaller in size, likely representing immature LBs. Although volumes of MVBs implicated in the shuttling of surfactant components either during recycling or de novo synthesis were unaltered, volumes of CBs were significantly increased suggesting an enhanced formation of surfactant precursors. This could be the result of an increased secretory activity of AE2 cells in the CD-Hyp group. Similar alterations have been observed upon stimulation of surfactant metabolism and secretion by surfactant instillation (Pinkerton et al. 1993). An increased consumption of surfactant under hypoxia might be due to a lower quality or quantity of the synthesized surfactant maybe due to a lack of appropriate amounts of synthetic precursors, and/or to a higher breathing activity. The lamellar body volume relative to the alveolar surface area was similar in CD-Hyp and CD implying comparable intracellular surfactant reserves. Nevertheless, mechanical changes in CD-Hyp demonstrated a reduction of pulmonary elastic recoil properties. It has been shown before that septal extracellular matrix changes in CD-Hyp are related to reduced elasticity H and increased lung compliance (Pankoke et al. 2023). The shift of inspiratory limbs of PV loops in CD-Hyp, however, indicates additional changes in surfactant function (Schipke et al. 2021). As a limitation of this study, we did not study isolated surfactants with regard to composition or function. This should be addressed in future experiments.

In the HSD-Hyp and HFD-Hyp groups, no ultrastructural signs of altered surfactant assembly and storage organelles were observed. Moreover, LB volumes per alveolar area were elevated compared to the corresponding normoxic groups, suggesting a compensatory increase in intracellular surfactant pools, possibly to meet the increased demand caused by the higher respiratory activity during hypoxia (Pankoke et al. 2023). Perhaps the diet-related lipid reserves in the AE2 cells provided sufficient lipid precursors for surfactant synthesis even during hypoxia, in contrast to the CD-Hyp mice. Moreover, inspiration limbs of PV loops were less shifted (HSD-Hyp) or almost unchanged (HFD-Hyp) in contrast to the CD-Hyp animals, indicating a more physiological surfactant function. These data could be interpreted as an obesity paradox: lipid accumulation can have a lipotoxic and thus adverse effect on the cell as reported before for inter alia the lung (Plantier et al. 2012; Tsai et al. 2021), but could also be a protective reserve in deficiency states, e.g., triggered by chronic diseases (Yao et al. 2023).

## Conclusions

Taken together, dietary sucrose and fat induce distinct metabolic states that influence adaptive responses of the pulmonary lipid metabolism and surfactant assembly in AE2 cells to chronic hypoxia. Thus, individual differences in diet and body weight may affect the susceptibility and the progression of lung diseases, and that should be taken into account for personalized prevention and therapy.

## Supplementary Information

Below is the link to the electronic supplementary material.

Supplementary file1 (DOCX 113 KB)

## Data Availability

The datasets used and/or analysed during the current study are available from the corresponding author on reasonable request.
