# Supplementary material for: Sucrose- and fat-related metabolic states influence the adaptation of the pulmonary lipid metabolism to hypoxia
Source: Cell Tissue Res. 2025 Apr 1;401(1):69–81. doi: 10.1007/s00441-025-03968-0 (PMC12222401; doi:10.1007/s00441-025-03968-0)
Supplement: Supplementary file 1 — Supplementary file1 (DOCX 113 KB) [file 441_2025_3968_MOESM1_ESM.docx]

**Data supplement**

**Sucrose- and fat-related metabolic states influence adaptation of the pulmonary lipid metabolism to hypoxia**

Sophia Pankoke^1,2^, Lea Gerling^2^, Matthias Ochs^3,4^, Christian Mühlfeld^2,5^, Julia Schipke^2,5^

^1^ Institute of Anatomy, University of Veterinary Medicine, Hannover, Germany

^2^ Hannover Medical School, Institute of Functional and Applied Anatomy, Carl-Neuberg-Str. 1, 30625 Hannover, Germany

^3^ Institute of Functional Anatomy, Charitè – Universitätsmedizin Berlin, Berlin, Germany

^4^ German Center for Lung Research (DZL), Berlin, Germany

^5^ Biomedical Research in Endstage and Obstructive Lung Disease, Member of the German Center for Lung Research (DZL), Hannover, Germany

**Corresponding author:**

PD Dr. Julia Schipke

Institute of Functional and Applied Anatomy, Hannover Medical School, Carl-Neuberg-Str. 1,

30625 Hannover, Germany

Tel.: +49 511 532 2997

E-Mail: [schipke.julia@mh-hannover.de](mailto:schipke.julia@mh-hannover.de)

**
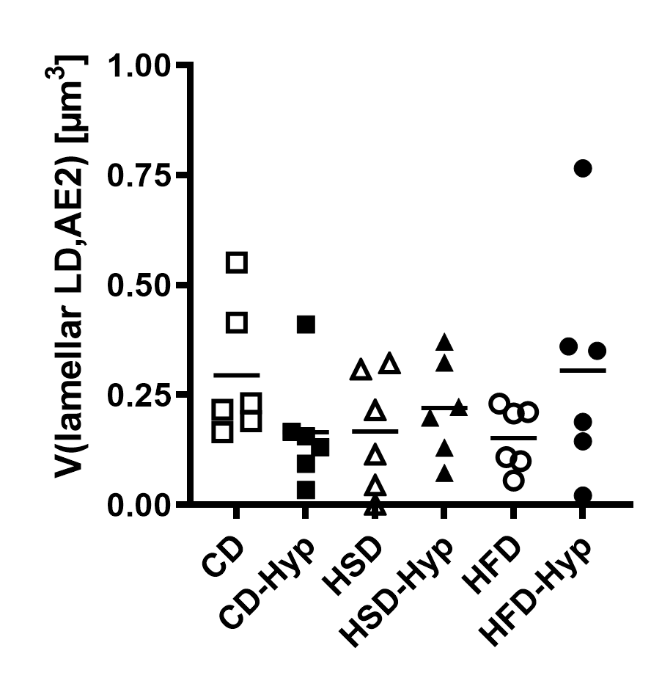
**

**Figure S1. Volumes of lamellated lipid droplets in AE2 cells.** Symbols represent values of individual mice, bars represent group means. Statistics: Data were compared by two-way ANOVA and post hoc Tukey test, no significant differences between groups were detected.

**Table S1. Reactome pathways of ANOVA-significant proteins according to STRING-db analysis.**

| **Reactome pathway** | **ANOVA proteins** | **background proteins** | **strength** | **FDR** |
| --- | --- | --- | --- | --- |
| Pyrophosphate hydrolysis | 2 | 3 | 1.58 | 0.038 |
| Beta oxidation of decanoyl-CoA to octanoyl-CoA-CoA | 3 | 6 | 1.46 | 0.0087 |
| ChREBP activates metabolic gene expression | 2 | 4 | 1.46 | 0.0499 |
| Biosynthesis of D-series resolvins | 2 | 4 | 1.46 | 0.0499 |
| Erythrocytes take up oxygen and release carbon dioxide | 3 | 7 | 1.39 | 0.0107 |
| Microtubule-dependent trafficking of connexons from Golgi to the plasma membrane | 5 | 15 | 1.28 | 0.00087 |
| GRB2:SOS provides linkage to MAPK signaling for Integrins | 5 | 15 | 1.28 | 0.00087 |
| Chylomicron assembly | 3 | 10 | 1.24 | 0.0207 |
| Erythrocytes take up carbon dioxide and release oxygen | 3 | 11 | 1.19 | 0.0241 |
| Signal regulatory protein family interactions | 3 | 11 | 1.19 | 0.0241 |
| p130Cas linkage to MAPK signaling for integrins | 4 | 15 | 1.18 | 0.0064 |
| Sealing of the nuclear envelope (NE) by ESCRT-III | 6 | 25 | 1.14 | 0.00064 |
| Degradation of cysteine and homocysteine | 3 | 13 | 1.12 | 0.0326 |
| RHO GTPases activate IQGAPs | 6 | 27 | 1.11 | 0.00087 |
| Other interleukin signaling | 3 | 15 | 1.06 | 0.0428 |
| Complex I biogenesis | 11 | 56 | 1.05 | 2.73E-06 |
| Mitochondrial Fatty Acid Beta-Oxidation | 7 | 37 | 1.04 | 0.0005 |
| Carboxyterminal post-translational modifications of tubulin | 5 | 26 | 1.04 | 0.0046 |
| Common Pathway of Fibrin Clot Formation | 4 | 21 | 1.04 | 0.0146 |
| Citric acid cycle (TCA cycle) | 4 | 22 | 1.02 | 0.0167 |
| Downstream signal transduction | 5 | 28 | 1.01 | 0.0057 |
| Aggrephagy | 6 | 35 | 0.99 | 0.002 |
| Gluconeogenesis | 5 | 33 | 0.94 | 0.0095 |
| Binding and Uptake of Ligands by Scavenger Receptors | 5 | 34 | 0.93 | 0.0104 |
| Plasma lipoprotein remodeling | 4 | 27 | 0.93 | 0.0269 |
| Respiratory electron transport | 14 | 96 | 0.92 | 1.73E-06 |
| COPI-independent Golgi-to-ER retrograde traffic | 7 | 48 | 0.92 | 0.0016 |
| ISG15 antiviral mechanism | 4 | 28 | 0.91 | 0.0295 |
| The role of GTSE1 in G2/M progression after G2 checkpoint | 10 | 73 | 0.9 | 0.00013 |
| Recycling pathway of L1 | 5 | 36 | 0.9 | 0.0121 |
| COPI-mediated anterograde transport | 13 | 95 | 0.89 | 5.12E-06 |
| HSP90 chaperone cycle for steroid hormone receptors (SHR) in the presence of ligand | 7 | 52 | 0.89 | 0.002 |
| The citric acid (TCA) cycle and respiratory electron transport | 21 | 167 | 0.86 | 5.28E-09 |
| Respiratory electron transport, ATP synthesis by chemiosmotic coupling, and heat production by uncoupling proteins. | 15 | 119 | 0.86 | 2.51E-06 |
| Metabolism of fat-soluble vitamins | 6 | 47 | 0.86 | 0.0067 |
| Pyruvate metabolism and Citric Acid (TCA) cycle | 6 | 48 | 0.86 | 0.0072 |
| Signaling by SCF-KIT | 5 | 40 | 0.86 | 0.0172 |
| MAP2K and MAPK activation | 5 | 40 | 0.86 | 0.0172 |
| Detoxification of Reactive Oxygen Species | 4 | 33 | 0.84 | 0.0443 |
| Cargo concentration in the ER | 4 | 33 | 0.84 | 0.0443 |
| Fatty acyl-CoA biosynthesis | 4 | 33 | 0.84 | 0.0443 |
| COPI-dependent Golgi-to-ER retrograde traffic | 11 | 94 | 0.83 | 0.00015 |
| ER-Phagosome pathway | 4 | 34 | 0.83 | 0.0475 |
| Retinoid metabolism and transport | 5 | 43 | 0.82 | 0.0212 |
| Nuclear Envelope (NE) Reassembly | 7 | 63 | 0.8 | 0.0051 |
| mRNA 3-end processing | 6 | 55 | 0.8 | 0.0117 |
| Kinesins | 6 | 55 | 0.8 | 0.0117 |
| ER to Golgi Anterograde Transport | 16 | 148 | 0.79 | 3.23E-06 |
| Peroxisomal protein import | 7 | 66 | 0.78 | 0.0062 |
| Cross-presentation of soluble exogenous antigens (endosomes) | 5 | 48 | 0.78 | 0.0295 |
| Response to elevated platelet cytosolic Ca2+ | 13 | 126 | 0.77 | 7.81E-05 |
| Golgi-to-ER retrograde transport | 13 | 128 | 0.77 | 8.71E-05 |
| L1CAM interactions | 7 | 68 | 0.77 | 0.007 |
| SRP-dependent cotranslational protein targeting to membrane | 9 | 90 | 0.76 | 0.0018 |
| Formation of the ternary complex, and subsequently, the 43S complex | 5 | 50 | 0.76 | 0.0336 |
| Platelet degranulation | 12 | 121 | 0.75 | 0.00024 |
| Antigen processing-Cross presentation | 9 | 91 | 0.75 | 0.0019 |
| Nonsense Mediated Decay (NMD) independent of the Exon Junction Complex (EJC) | 9 | 92 | 0.75 | 0.002 |
| Intra-Golgi and retrograde Golgi-to-ER traffic | 18 | 186 | 0.74 | 2.73E-06 |
| Integrin cell surface interactions | 7 | 73 | 0.74 | 0.0095 |
| RNA Polymerase II Transcription Termination | 6 | 64 | 0.73 | 0.0207 |
| Intraflagellar transport | 5 | 53 | 0.73 | 0.0409 |
| Fatty acid metabolism | 15 | 165 | 0.72 | 5.32E-05 |
| Hedgehog off state | 10 | 108 | 0.72 | 0.0015 |
| L13a-mediated translational silencing of Ceruloplasmin expression | 10 | 109 | 0.72 | 0.0016 |
| GTP hydrolysis and joining of the 60S ribosomal subunit | 10 | 110 | 0.72 | 0.0016 |
| Formation of a pool of free 40S subunits | 9 | 99 | 0.72 | 0.0029 |
| Nuclear events mediated by NFE2L2 | 5 | 54 | 0.72 | 0.0428 |
| Nonsense Mediated Decay (NMD) enhanced by the Exon Junction Complex (EJC) | 10 | 112 | 0.71 | 0.0017 |
| Iron uptake and transport | 5 | 56 | 0.71 | 0.0472 |
| Post-translational protein phosphorylation | 10 | 115 | 0.7 | 0.002 |
| Protein localization | 9 | 104 | 0.7 | 0.004 |
| Dectin-1 mediated noncanonical NF-kB signaling | 5 | 57 | 0.7 | 0.0499 |
| NIK-->noncanonical NF-kB signaling | 5 | 57 | 0.7 | 0.0499 |
| Translation initiation complex formation | 5 | 57 | 0.7 | 0.0499 |
| Ribosomal scanning and start codon recognition | 5 | 57 | 0.7 | 0.0499 |
| Transport of Mature mRNA derived from an Intron-Containing Transcript | 6 | 70 | 0.69 | 0.0279 |
| Signaling by MET | 6 | 70 | 0.69 | 0.0279 |
| Regulation of Insulin-like Growth Factor (IGF) transport and uptake by Insulin-like Growth Factor Binding Proteins (IGFBPs) | 10 | 121 | 0.68 | 0.0025 |
| ABC-family proteins mediated transport | 8 | 99 | 0.67 | 0.0104 |
| RHOG GTPase cycle | 6 | 73 | 0.67 | 0.0326 |
| Metabolism of vitamins and cofactors | 13 | 168 | 0.65 | 0.00077 |
| Signaling by Hedgehog | 11 | 141 | 0.65 | 0.002 |
| CLEC7A (Dectin-1) signaling | 7 | 89 | 0.65 | 0.0212 |
| Downstream signaling events of B Cell Receptor (BCR) | 6 | 77 | 0.65 | 0.0402 |
| FCERI mediated NF-kB activation | 6 | 77 | 0.65 | 0.0402 |
| Glucose metabolism | 6 | 79 | 0.64 | 0.0429 |
| C-type lectin receptors (CLRs) | 8 | 110 | 0.62 | 0.0171 |
| Asparagine N-linked glycosylation | 18 | 264 | 0.59 | 0.00016 |
| Platelet activation, signaling and aggregation | 17 | 250 | 0.59 | 0.00027 |
| Signaling by the B Cell Receptor (BCR) | 7 | 103 | 0.59 | 0.0386 |
| Fc epsilon receptor (FCERI) signaling | 8 | 121 | 0.58 | 0.0255 |
| Cellular response to chemical stress | 10 | 159 | 0.56 | 0.0129 |
| Toll-like Receptor Cascades | 9 | 143 | 0.56 | 0.0207 |
| MHC class II antigen presentation | 8 | 126 | 0.56 | 0.0308 |
| Vesicle-mediated transport | 37 | 592 | 0.55 | 2.58E-08 |
| Signaling by Interleukins | 16 | 262 | 0.54 | 0.0012 |
| Separation of Sister Chromatids | 11 | 183 | 0.54 | 0.0107 |
| Neutrophil degranulation | 31 | 526 | 0.53 | 2.51E-06 |
| Metabolism of amino acids and derivatives | 15 | 251 | 0.53 | 0.002 |
| Mitotic Anaphase | 13 | 219 | 0.53 | 0.0051 |
| Membrane Trafficking | 32 | 559 | 0.52 | 2.51E-06 |
| Hemostasis | 28 | 501 | 0.51 | 1.75E-05 |
| Extracellular matrix organization | 14 | 247 | 0.51 | 0.0048 |
| Metabolism | 96 | 1754 | 0.5 | 3.13E-20 |
| Translation | 12 | 225 | 0.49 | 0.0152 |
| Innate Immune System | 50 | 945 | 0.48 | 5.28E-09 |
| Axon guidance | 14 | 268 | 0.48 | 0.0087 |
| Cellular responses to stress | 23 | 448 | 0.47 | 0.00048 |
| Cytokine Signaling in Immune system | 20 | 397 | 0.46 | 0.0016 |
| MAPK1/MAPK3 signaling | 14 | 277 | 0.46 | 0.0107 |
| MAPK family signaling cascades | 15 | 302 | 0.45 | 0.0089 |
| RAF/MAP kinase cascade | 13 | 271 | 0.44 | 0.0212 |
| Immune System | 74 | 1615 | 0.42 | 4.25E-11 |
| Signaling by Rho GTPases, Miro GTPases and RHOBTB3 | 28 | 618 | 0.41 | 0.00048 |
| Metabolism of lipids | 27 | 596 | 0.41 | 0.00059 |
| Transport of small molecules | 29 | 658 | 0.4 | 0.0005 |
| Signaling by Receptor Tyrosine Kinases | 18 | 410 | 0.4 | 0.0097 |
| Signaling by Rho GTPases | 26 | 603 | 0.39 | 0.0015 |
| Adaptive Immune System | 28 | 687 | 0.37 | 0.0017 |
| RHO GTPase cycle | 17 | 422 | 0.36 | 0.0243 |
| Metabolism of proteins | 61 | 1616 | 0.34 | 2.73E-06 |
| Metabolism of RNA | 23 | 599 | 0.34 | 0.0103 |
| Post-translational protein modification | 46 | 1269 | 0.32 | 0.00025 |
| Signal Transduction | 68 | 2212 | 0.25 | 0.00027 |

**Table S2. Lipid-metabolism associated proteins with significantly altered abundances due to experimental conditions.**

| **Gene name** | **Protein name** | **Log_10_ p-value** | **Log_2_ fold-change** |
| --- | --- | --- | --- |
| **CD-Hyp** | | | |
| **Hpgd** | 15-hydroxyprostaglandin dehydrogenase [NAD(+)] | 2.26 | -1.65 |
| Mecr | Trans-2-enoyl-CoA reductase, mitochondrial | 2.53 | -1.52 |
| Cyp4b1 | Cytochrome P450 4B1 | 1.74 | -0.72 |
| **Alb** | Serum albumin | 1.49 | -0.42 |
| **Idi1** | Isopentenyl-diphosphate Delta-isomerase 1 | 1.63 | 0.57 |
| Degs1 | Sphingolipid delta(4)-desaturase DES1 | 1.46 | 0.58 |
| Agps | Alkyldihydroxyacetonephosphate synthase, peroxisomal | 1.60 | 0.85 |
| Csnk2a1 | Casein kinase II subunit alpha | 1.70 | 1.28 |
| **HSD-Hyp** | | | |
| **Hsd17b8** | Estradiol 17-beta-dehydrogenase 8 | 2.14 | -1.73 |
| Hmgcl | Hydroxymethylglutaryl-CoA lyase, mitochondrial | 1.57 | -1.66 |
| Decr1 | 2,4-dienoyl-CoA reductase, mitochondrial | 4.80 | -1.38 |
| Acad10 | Acyl-CoA dehydrogenase family member 10 | 1.35 | -1.23 |
| Plpp1/ Ppap2a | Lipid phosphate phosphohydrolase 1 | 3.80 | -1.13 |
| **Hpgd** | 15-hydroxyprostaglandin dehydrogenase [NAD(+)] | 3.70 | -1.07 |
| **Cbr1** | Carbonyl reductase [NADPH] 1 | 4.01 | -0.99 |
| Pitpnb | Phosphatidylinositol transfer protein beta isoform | 1.33 | -0.65 |
| Hadh | Hydroxyacyl-coenzyme A dehydrogenase, mitochondrial | 2.25 | -0.55 |
| **Alb** | Serum albumin | 1.69 | -0.49 |
| Gc | Vitamin D-binding protein | 1.49 | -0.46 |
| Acaa2 | 3-ketoacyl-CoA thiolase, mitochondrial | 1.42 | -0.37 |
| Hadhb | Trifunctional enzyme subunit beta, mitochondrial;3-ketoacyl-CoA thiolase | 1.46 | 0.48 |
| Lta4h | Leukotriene A-4 hydrolase | 1.55 | 0.50 |
| Hsd11b1 | Corticosteroid 11-beta-dehydrogenase isozyme 1 | 1.31 | 0.58 |
| **Idi1** | Isopentenyl-diphosphate Delta-isomerase 1 | 1.90 | 0.63 |
| Sacm1l | Phosphatidylinositide phosphatase SAC1 | 2.79 | 0.66 |
| Lpcat1 | Lysophosphatidylcholine acyltransferase 1 | 2.41 | 0.79 |
| Slc44a1 | Choline transporter-like protein 1 | 2.13 | 0.89 |
| Gpx4 | Phospholipid hydroperoxide glutathione peroxidase, mitochondrial | 2.46 | 0.95 |
| Hadha | Trifunctional enzyme subunit alpha, mitochondrial;Long-chain enoyl-CoA hydratase;Long chain 3-hydroxyacyl-CoA dehydrogenase | 2.81 | 0.96 |
| Agpat4 | 1-acyl-sn-glycerol-3-phosphate acyltransferase delta | 1.87 | 1.04 |
| Pi4ka | Phosphatidylinositol 4-kinase alpha | 1.63 | 1.23 |
| Acot11 | Acyl-coenzyme A thioesterase 11 | 1.64 | 1.29 |
| Pik3r1 | Phosphatidylinositol 3-kinase regulatory subunit alpha | 1.68 | 1.34 |
| Rufy1 | RUN and FYVE domain-containing protein 1 | 2.17 | 1.60 |
| **HFD-Hyp** | | | |
| **Hpgd** | 15-hydroxyprostaglandin dehydrogenase [NAD(+)] | 4.38 | -1.89 |
| **Hsd17b8** | Estradiol 17-beta-dehydrogenase 8 | 1.61 | -1.15 |
| Cbr4 | Carbonyl reductase family member 4 | 2.18 | -1.12 |
| Inpp5d | Phosphatidylinositol 3,4,5-trisphosphate 5-phosphatase 1 | 1.32 | -1.08 |
| **Cbr1** | Carbonyl reductase [NADPH] 1 | 1.38 | -0.56 |
| Plbd1 | Phospholipase B-like 1; chain A, B, C | 1.35 | 0.38 |
| Mgll | Monoglyceride lipase | 1.51 | 0.86 |
| Fabp5 | Fatty acid-binding protein, epidermal | 1.40 | 0.99 |
| Stard5 | StAR-related lipid transfer protein 5 | 1.76 | 1.52 |
| Pmvk | Phosphomevalonate kinase | 2.07 | 2.30 |

Proteins also significantly regulated in at least one of the other experimental groups are shown in bold.
